# Insulin Resistance and Cognitive Function in Nondiabetic Patients With Cerebral Small Vessel Disease: Role of Brain Glymphatic Function

**DOI:** 10.1002/brb3.71143

**Published:** 2025-12-22

**Authors:** Xiao‐Li Yang, Yu‐Fan Luo, Tian‐Yao Wang, Meng‐Xiang Wang, Wen‐Mei Lu, Hu‐Lie Zeng, Dan‐Hong Wu

**Affiliations:** ^1^ Department of Neurology Shanghai Fifth People's Hospital Fudan University Shanghai China; ^2^ Department of Radiology Renji Hospital Shanghai Jiao Tong University School of Medicine Shanghai China; ^3^ Key Laboratory of Smart Drug Delivery School of Pharmacy Ministry of Education Fudan University Shanghai China; ^4^ Joint Center for Translational Medicine Shanghai Fifth People's Hospital Fudan University and School of Life Science East China Normal University Shanghai China; ^5^ Center of Community‐Based Health Research Fudan University Shanghai China

**Keywords:** brain glymphatic function, cerebral small vessel disease, cognitive function, diffusion tensor imaging along the perivascular space (DTI‐ALPS) index, insulin sensitivity

## Abstract

**Objective:**

Both insulin sensitivity and brain glymphatic function were linked to cognitive function in nondiabetic patients with cerebral small vessel disease (CSVD). This study aims to elucidate these complex associations through mediation analysis.

**Methods:**

Patients who underwent multimodal magnetic resonance imaging (MRI) scans were recruited. Global cognitive assessment was performed by Mini‐Mental State Examination (MMSE), four isolated cognitive domains were assessed simultaneously. Glymphatic function was assessed using the diffusion tensor imaging along the perivascular space (DTI‐ALPS) index, insulin sensitivity was assessed by the homeostasis model assessment–estimated insulin resistance index (HOMA‐IR), and HOMA‐IR≥2.80 was defined as insulin resistance (IR). Mediation analysis was conducted to examine the effect of the DTI‐ALPS on insulin sensitivity and cognitive function.

**Results:**

110 CSVD patients were recruited, 40 patients were IR and 70 patients were non‐IR. Both the HOMA‐IR and DTI‐ALPS index were significant predictors of cognitive function, with B = −0.592, 95%CI, −0.939 to −0.244, *p* = 0.001 and B = 9.378, 95% CI, 3.376 to 15.381, *p* = 0.003, respectively, after adjusting for age, sex, and other confounding factors. Mediation analysis revealed that the DTI‐ALPS index served as a significant partial mediator in the relationship between HOMA‐IR and cognitive function, with direct effect = −0.649 (95% CI, −1.015 to −0.282, *p* < 0.001), total effect = −0.743 (95% CI, −1.106 to −0.380, *p* < 0.001), and the indirect effect = −0.094 (95% CI, −0.236 to −0.006, *p* = 0.029), accounting for 12.66% of the total effect.

**Conclusion:**

Both the HOMA‐IR and the DTI‐ALPS index are independent risk factors for cognitive function. Furthermore, the DTI‐ALPS index significantly and partially mediates the relationship between the HOMA‐IR and cognitive function.

## Graphical abstract

The DTI‐ALPS index partially mediates the association of insulin resistance (lR), assessed by HOMA‐IR, with cognitive function, with both identified as independent risk factors.

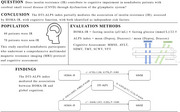

**Abbreviations**
AQP‐4: aquaporin‐4
AVLT: auditory verbal learning test
BBB: blood–brain barrier
CSF: cerebrospinal fluid
CSVD: cerebral small vessel disease
DTI‐ALPS index: diffusion tensor imaging along the perivascular space index
EPVS: enlarged perivascular spaces
FLAIR: fluid‐attenuated inversion recovery
HDL: high‐density lipoprotein
HOMA‐IR: homeostasis model assessment‐insulin resistance
hsCRP: high‐sensitivity C‐reactive protein
IR: insulin resistance
ISCDECSHAP: Investigation on the Status of Cerebrovascular Diseases and Establishing Cohort in Shang Hai Aging Population
LDL: low‐density lipoprotein
MMSE: Mini‐Mental State Examination
MRI: magnetic resonance imaging
NGVU: neuro‐glial‐vascular units
PVS: perivascular spaces
SCWT‐C: stroop color‐word test‐C
SDMT: symbol digit modalities test
SWI: susceptibility‐weighted imaging
tCSVD score: total cerebral small vessel disease score
TMT‐B: trails making test‐B
VFT: verbal fluency test
WMH: white matter hyperintensity.

## 1 Background

Cerebral small vessel disease (CSVD) describes heterogeneous conditions affecting the blood vessels of 50 to 500 µm in diameter (Duering et al. 2023). To be the most common cerebrovascular diseases in the elderly, CSVD may be presented as stroke, cognitive impairment, and psychiatric disturbance (Duering et al. 2023). Epidemiological studies have found that CSVD contributed 20% to 40% of dementia, and increased 75% the risk of developing vascular dementia (Hamilton et al. 2021; Rundek et al. 2022). Dysfunction of the “vascular–neural–cognitive” axis constitutes the core mechanism underlying CSVD‐related cognitive impairment (Kremer et al. 2025), but how metabolic factors exert their influence within this axis remains to be fully elucidated.

The core structure of the “vascular‐neural‐cognitive” axis between neural activity and cerebral blood flow, composed of vascular cells, glia, neurons, and the basal lamina matrix within the brain vasculature, is namely “neuro‐glial‐vascular units (NGVU)”. Growing evidence indicates a significant association between neuro‐glial‐vascular unit dysfunction and CSVD (Kremer et al. 2025). As one of the key components of NGVU, the brain glymphatic function mediate the rapid exchange of cerebrospinal fluid (CSF) and interstitial fluid, with significant functions in maintaining homeostasis of the central nervous system (Aspelund et al. 2015; Louveau et al. 2015). Intrathecal administration of gadolinium‐based MRI tracers is currently the gold standard for assessing glymphatic function in humans (Taoka and Naganawa 2020), while advancements in neuroimaging have shown that the ALPS index, derived from DTI, is highly correlated with this gold standard and can effectively gauge glymphatic clearance capacity (Taoka et al. 2017).

Insulin resistance (IR), a hallmark of metabolic syndrome, is defined as reduced sensitivity to the action of insulin, which impairs microvascular endothelium and disrupts the blood–brain barrier (BBB) by inhibiting the nitric‐oxide pathway, provoking low‐grade inflammation, and triggering oxidative stress (Nakhaee et al. 2024). Thus, IR may be related to NGVU closely (Lee et al. 2022; van Sloten et al. 2020). Previous studies have demonstrated that IR was related to total CSVD neuroimaging burden and cognitive function in nondiabetic patients with CSVD (Cui et al. 2022; Yang et al. 2019). In addition, emerging clinical studies found that impairment of the glymphatic clearance function was associated with increased severity of IR (Yang et al. 2020), and patients with IR had a significantly lower DTI‐ALPS index than the healthy controls (Andica et al. 2023). Therefore, we speculated that IR, a metabolic factor, plays a pivotal role in NGVU function by modulating the glymphatic system. To confirm our hypothesis, we applied the DTI‐ALPS index to assess the functioning of the brain glymphatic function, the homeostasis model assessment‐insulin resistance (HOMA‐IR) to assess the insulin sensitivity, and to explore the relationship between IR and cognitive function from the perspective of glymphatic function in nondiabetic patients with CSVD.

## 2 Methods

### 2.1 Study Design and Population

“Investigation on the Status of Cerebrovascular Diseases and Establishing Cohort in Shang Hai Aging Population (ISCDECSHAP)” is a prospective, population‐based, and cohort study of stroke incidence and risk factors in an aging population from Shang Hai City. The ISCDECSHAP study aimed to establish a Chinese CSVD community cohort, and was approved by the ethics committee of Shanghai Fifth People's Hospital. Written informed consent was obtained from all the patients or their representatives before data collection. Based on protocol, all the subjects, at least 60 years old, performed cerebral magnetic resonance imaging (MRI), carotid artery ultrasound, cognitive function, and hematologic examination. All the examinations of this study were completed within a week. CSVD was diagnosed in participants who exhibited: (1) moderate‐to‐severe white‐matter lesions (WMH) (deep Fazekas score > 1 or periventricular Fazekas score > 2); or (2) mild WMH (deep Fazekas score = 1 or periventricular Fazekas score = 2) together with at least one lacunar infarct or three or more cerebral microbleeds. We only recruited nondiabetic patients with CSVD from this population into our study. Patients who met any one of the following criteria were excluded: history of cortical or watershed infarcts, intracerebral hemorrhage, hydrocephalus, or white‐matter lesions attributable to other established etiologies (such as multiple sclerosis, metabolic disorders, or toxic causes).

### 2.2 Baseline Characteristics Collection

Baseline demographic characteristics, including age, sex, and comorbid conditions were obtained through face‐to‐face interviews with the neurological resident. Fasting venous blood samples were used to measure glucose, insulin, and other laboratory examinations. Insulin sensitivity was evaluated by the HOMA‐IR, which was calculated as fasting insulin (µU/mL) × fasting glucose (mmol/L)/22.5 (Matthews et al. 1985). Referring to previous researches (Jhuang et al. 2019; Yang et al. 2019), HOMA‐IR ≥2.80 was defined as IR.

Global cognition was evaluated with the Chinese version of the Mini‐Mental State Examination (MMSE) (Li et al. 2016). Four isolated cognitive domains were assessed, including verbal memory, attention, language, and executive function. To measure verbal memory, we administered the Chinese Huashan version of the Auditory Verbal Learning Test (Zhao et al. 2012). Three indices were recorded: AVLT‐learning (sum of words recalled across the first three immediate‐recall trials), AVLT‐recall (delayed recall after 20 min), and AVLT‐recognition. Attention was assessed with the Symbol Digit Modalities Test (SDMT). Executive function was evaluated with the Trails Making Test‐B (TMT‐B) and Stroop Color‐Word Test‐C (SCWT‐C). Language function was examined with the Verbal Fluency Test (VFT). Longer completion times on TMT‐B and SCWT‐C indicate poorer performance, whereas higher scores on AVLT, SDMT, and VFT indicate better performance. Composite z‐scores were then created for global cognition, memory, attention, executive, and language functioning by averaging the z‐transformed data of individual tests. The composite z‐scores were used to validate differences between groups.

### 2.3 Brain MRI and the Total CSVD Score

Neuroimaging examinations were performed using a 3.0T MRI system with a standard 8‐channel HRBRAIN coil. Axial T2‐weighted sequences, fluid‐attenuated inversion recovery (FLAIR), T1‐weighted sequences, susceptibility‐weighted imaging (SWI), and DTI were used for image review. The specific parameter information for MRI acquisition was shown in Supplementary Materials. Individual imaging features of CSVD were observed strictly by Neuroimaging standards (Duering et al. 2023). The Fazekas scale was used for periventricular and deep WMH evaluation (Fazekas et al. 1987). Lacune and microbleeds were numbered. Enlarged perivascular spaces (EPVS) were rated as 3 grades in the basal ganglia manually based on semi‐quantitative scales (1 grade, the number is 0–10; 2 grade, the number is 11–25; and 3 grade, the number >25) (Yang et al. 2019). The total CSVD score was calculated to capture the overall effect of CSVD on the brain as follows (Staals et al. 2014), with 1 point being awarded for each of these features: periventricular Fazekas score = 3, or deep Fazekas score ≥2, for WMH; 1 or more lacunes; 1 or more microbleeds; and grade 2–3 for EPVS in the basal ganglia. Therefore, the total CSVD score was stratified from 0 to 4. The MRIs were independently evaluated by two neurologists, and inter‐observer agreement values for the presence of microbleed, WMH, EPVS, and lacune were 0.80, 0.83, 0.78, and 0.79, respectively. Any disagreement regarding the presence of CSVD features was resolved by consensus with the third neuroimaging expert.

### 2.4 Assessment of Brain Glymphatic Function by the DTI‐ALPS Index

The activity of the brain's glymphatic function was indirectly assessed using the DTI‐ALPS methodology (Supplementary Figure 1). DTI data were processed using FSL 6.0.1 (FMRIB Software Library https://fsl.fmrib.ox.ac.uk/fsl) to generate fractional anisotropy (FA) maps and directional diffusivity maps (Dxx, Dyy, Dzz) along three orthogonal axes. Specifically: (a) Projection fibers adjacent to the lateral ventricles predominantly followed the z‐axis orientation. (b) Association fibers aligned with the y‐axis trajectory. (c) Subcortical fibers exhibited parallel orientation to perivascular spaces (PVS) along the x‐axis. Six spherical regions of interest (ROIs) with a diameter of 5 mm were defined at the bilateral ventricle body level in MNI152 standard space, targeting three bilateral fiber groups: projection fibers (Dxproj, Dyproj), association fibers (Dxassoc, Dzassoc), and subcortical fibers. Axial diffusivity analysis revealed that x‐axis diffusivity in projection (Dxproj) and association fibers (Dxassoc) primarily captured PVS‐mediated water mobility with minimal fiber tract contamination. In contrast, y‐axis projection fiber diffusivity (Dyproj) and z‐axis association fiber diffusivity (Dzassoc) represented non‐PVS directional water diffusion. The divergence between x‐axis diffusivity parameters (Dxproj, Dxassoc) and composite y/z‐axis diffusivity measurements (Dyproj, Dzassoc) was interpreted as reflecting PVS‐specific hydrodynamic characteristics. All diffusivity maps were registered into MNI152 standard space via 3D T1 images using ANTs (v2.3, https://github.com/ANTsX/ANTs). The DTI‐ALPS index was calculated using the formula: ALPS index = mean (Dxproj, Dxassoc) / mean (Dyproj, Dzassoc). Average DTI‐ALPS is calculated as the mean of the left and right DTI‐ALPS indices. The workflow and illustration for DTI‐ALPS are shown in Supplementary Figure 2.

### 2.5 Statistical Analysis

Statistical analyses were performed using SPSS 22. For demographic and clinical features, continuous variables with normal distribution were presented as mean ± standard deviation, and those with abnormal distribution were presented as median (interquartile range). Student t‐tests or ANOVAs for normally distributed continuous parameters and Wilcoxon or Kruskal–Wallis tests for abnormally distributed continuous parametric variables were used when appropriate. Categorical variables were expressed as frequency (percentage), and the χ2 test or Fisher exact test was used.

Spearman's correlation analysis was employed to measure bivariate correlations between HOMA‐IR, cognitive function, and the activity of the brain glymphatic function. Multivariate linear regression was used to explore the association between HOMA‐IR, DTI‐ALPS index and cognitive function. To examine the potential mediating role of the DTI‐ALPS index on the relationship between HOMA‐IR and cognitive function, mediation analyses were performed using PROCESS v4.1 in SPSS by Andrew F. Hayes (Coutts and Hayes 2023), after adjusting for age, sex, education level, and other confounding factors. Sobel and Bootstrap tests with 5000 iterations were used to examine the significance of the mediation models. All statistical tests were two‐sided, and *p* < 0.05 was considered statistically significant.

## 3 Results

### 3.1 Baseline Characteristics

Among 256 patients, 56 patients with unavailable or unqualified DTI data, and 90 patients with diabetes mellitus were excluded, and we enrolled 110 patients in this study finally (Supplementary Figure 2), among them, 40 patients with IR and 70 patients with non‐IR. The demographic and laboratory characteristics are shown in Table 1. No significant group differences in sex, education years, smoking, and drinking. The rate of CSVD burden with 3 and 4 was 22.50% and 25.00%, respectively, higher than 2.86% and 5.71% in the non‐IR group. In addition, compared with the non‐IR group, there was a lower DTI‐ALPS index and global cognition in patients with the IR group. Regarding various cognitive domains, the IR group demonstrated significant impairments in memory and executive function compared with the non‐IR group, with no significant differences observed in language and attention function between the two groups (Table 1).

**TABLE 1:** Baseline characteristics of the 110 participants.

Variables	Non‐IR	IR	P value
	N = 70	N = 40	
**Demographic characteristics**			
Male, n (%)	28(40.00)	18(45.00)	0.689
Education, median(Q25, Q75), years	12(9,12)	12(9,12)	0.981
Age, median(Q25, Q75), years	65(62.00, 69.00)	68(63.75, 73.25)	0.041
Smoking, n (%)	14(20.00)	9(22.50)	0.810
Drinking, n (%)	19(27.143)	11(27.500)	0.968
Hypertension, n (%)	33(47.143)	29(72.500)	0.016
**Laboratory examinations**			
Total cholesterol, mean (sd), mmol/L	4.57(1.004)	4.59(0.945)	0.923
Triglyceride, median(Q25, Q75), mmol/L	1.33(1.05, 1.61)	1.39(1.00, 1.63)	0.932
High‐density lipoprotein, mean (sd), mmol/L	1.41(0.40)	1.31(0.30)	0.173
Low‐density lipoprotein, mean (sd), mmol/L	2.95(0.90)	3.04(0.92)	0.646
Homocysteine, median(Q25, Q75), mmol/L	12.25(10.13, 14.18)	12.55(10.38, 14.25)	0.981
hsCRP, median(Q25, Q75), mg/L	0.66(0.30, 1.19)	1.27(0.42, 2.59)	0.008
Fasting blood glucose, mean (sd), mmol/L	5.28(0.90)	5.96(1.24)	0.001
Fasting insulin, mean (sd), µU/mL	5.76(2.75)	14.99(6.75)	<0.001
HOMA‐IR, mean (sd)	1.60(0.77)	4.59(1.82)	<0.001
**Neuroimaging**			
tCSVD score, n (%)			<0.001
0	23(32.86)	6(15.00)	
1	26(37.14)	7(17.50)	
2	15(21.43)	8(20.00)	
3	2(2.86)	9(22.50)	
4	4(5.71)	10(25.00)	
ALPS, mean (sd)	1.28(0.10)	1.21(0.10)	<0.001
**Cognitive function (z‐scores)**			
MMSE, mean (sd)	28.09(2.29)	25.58(4.65)	<0.001
**Verbal memory**			
AVLT N1‐3 (learning), mean (sd)	0.13(1.04)	−0.23(0.90)	0.071
AVLT N4 (recall), mean (sd)	0.18(1.02)	−0.31(0.89)	0.012
AVLT N5 (recognition), mean (sd)	0.14(1.10)	−0.25(0.74)	0.05
**Attention**			
SDMT, mean (sd)	0.10(0.98)	−0.23(1.01)	0.093
**Executive function**			
SCWT‐C, mean (sd)	−0.12(0.95)	0.30(1.02)	0.032
TMT‐B, mean (sd)	−0.07(0.92)	0.14(1.09)	0.299
**Language function**			
VFT, mean (sd)	0.66(1.02)	−0.09(0.98)	0.427

### 3.2 The Relationship Between Activity of the Brain Glymphatic Function, Insulin Sensitivity and Cognitive Function

**FIGURE 1:**
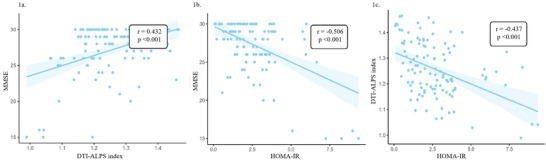
The relationship between activity of the brain glymphatic function, HOMA‐IR, and cognitive function. (a) The relationship between MMSE and DTI‐ALPS index, (b) the relationship between MMSE and HOMA‐IR, and (c) the relationship between HOMA‐IR and DTI‐ALPS index. Abbreviations: DTI‐ALPS index, diffusion tensor imaging along the perivascular space index; HOMA‐IR, homeostatis model assessment‐insulin resistance; MMSE, Mini‐Mental State Examination.

Spearman correlation analysis revealed significant inter‐correlations among the DTI‐ALPS index, HOMA‐IR, and MMSE (all *p* < 0.001). As illustrated in Figure 1, MMSE scores were positively correlated with the DTI‐ALPS index (r = 0.432, *p* < 0.001; Figure 1a) and negatively correlated with HOMA‐IR (r = −0.506, *p* < 0.001; Figure 1b). Conversely, HOMA‐IR was inversely associated with the DTI‐ALPS index (r = −0.437, *p* < 0.001; Figure 1c). After adjustment for age, hypertension, tCSVD burden, and other covariates, HOMA‐IR remained independently and inversely associated with the DTI‐ALPS index (B = −0.014, 95% CI, −0.026 to −0.003, *p* = 0.016; Table 2).

**TABLE 2:** The associations of HOMA‐IR and DTI‐ALPS index in non‐diabetic patients with CSVD.

Variable	DTI‐ALPS index	
	B(95%CI)	*p*
Male	−0.021(−0.065, 0.022)	0.331
Education	0.003(−0.004, 0.01)	0.420
Age	−0.004(−0.007, 0)	0.042
Smoking	−0.017(−0.073, 0.04)	0.562
Drinking	0.023(−0.023, 0.07)	0.325
Hypertension	−0.034(−0.075, 0.007)	0.099
Total cholesterol	−0.024(−0.094, 0.046)	0.505
Total triglyceride	0.006(−0.018, 0.03)	0.639
HDL	0.028(−0.042, 0.097)	0.432
LDL	0.003(−0.07, 0.075)	0.944
HCY	0.002(−0.001, 0.005)	0.217
hsCRP	−0.005(−0.014, 0.004)	0.292
HOMA‐IR	−0.014(−0.026, −0.003)	0.016
tCSVD score	−0.014(−0.033, 0.006)	0.162

We further explored the associations of HOMA‐IR, DTI‐ALPS index, and cognitive function by multifactor regression analysis (Table 3). HOMA‐IR was a significant predictor of cognitive function (B = −0.724, *p* < 0.001) after adjusting for age, sex, education level, hypertension, LDL, tCSVD score, and other confounding factors. This relationship remained significant when the DTI‐ALPS index was considered (B = −0.592, *p* = 0.001). Second, the DTI‐ALPS index correlated positively with cognitive function after adjusting for other confounding factors, including IR, with B = 9.378, 95% CI, 3.376, 15.381, *p* = 0.003.

**TABLE 3:** The associations of HOMA‐IR, DTI‐ALPS index and cognitive function.

Variable	MMSE (Model 1)		MMSE (Model 2)	
	B(95%CI)	*p*	B(95%CI)	*p*
Male	0.585(−0.753, 1.924)	0.388	0.786(−0.502, 2.074)	0.229
Education	0.285(0.056, 0.514)	0.015	0.257(0.037, 0.477)	0.023
Age	−0.103(−0.208, 0.001)	0.053	−0.07(−0.172, 0.032)	0.177
Smoking	1.785(0.044, 3.526)	0.045	1.941(0.271, 3.611)	0.023
Drinking	−0.359(−1.788, 1.069)	0.619	−0.576(−1.951, 0.798)	0.407
Hypertension	1.251(−0.012, 2.514)	0.052	1.574(0.347, 2.801)	0.012
Total cholesterol	0.723(−1.432, 2.878)	0.507	0.944(−1.124, 3.012)	0.367
Total triglyceride	0.23(−0.514, 0.974)	0.541	0.176(−0.537, 0.89)	0.625
HDL	0.778(−1.366, 2.923)	0.473	0.518(−1.542, 2.578)	0.619
LDL	−0.675(−2.893, 1.543)	0.547	−0.699(−2.823, 1.425)	0.515
HCY	−0.058(−0.157, 0.04)	0.244	−0.077(−0.172, 0.018)	0.112
hsCRP	0.216(−0.064, 0.497)	0.129	0.262(−0.008, 0.532)	0.057
HOMA‐IR	−0.724(−1.076, −0.372)	<0.001	−0.592(−0.939, −0.244)	0.001
tCSVD score	−0.464(−1.053, 0.125)	0.121	−0.337(−0.907, 0.233)	0.244
DTI‐ALPS	—		9.378(3.376, 15.381)	0.003

*Note*: Model 2 = Model 1 + DTI‐ALPS index.

### 3.3 The Mediating Role of Brain Glymphatic Function in the Relationship Between Insulin Sensitivity and Global Cognitive Function

**FIGURE 2:**
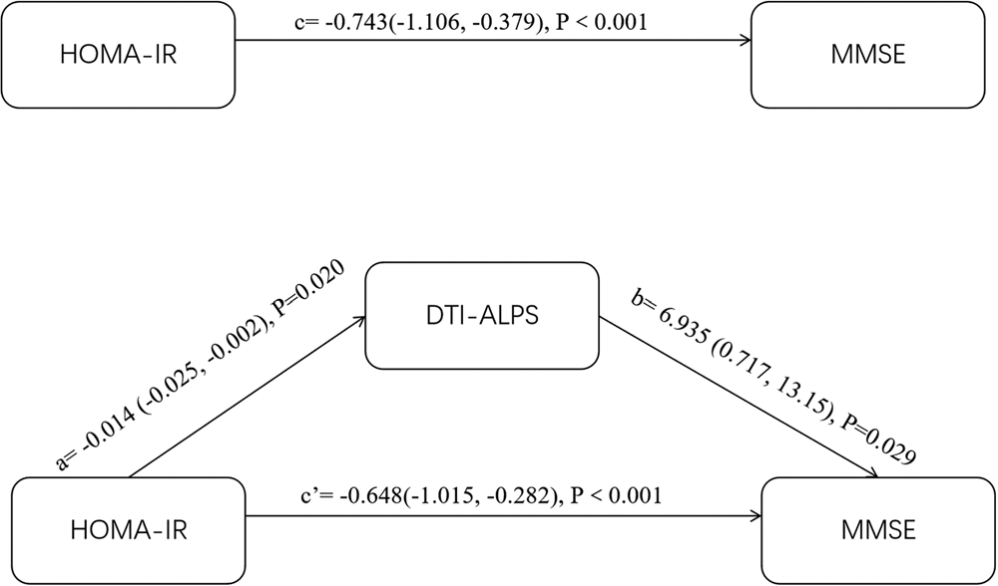
The mediating role of brain glymphatic function in the relationship between HOMA‐IR and global cognitive function.

Mediation analysis revealed that the DTI‐ALPS index served as a significant partial mediator in the relationship between HOMA‐IR and cognitive function, after controlling for covariates including age, sex, educational level, smoking status, hypertension, diabetes, tCSVD score, and laboratory examinations. To validate mediation effects, Sobel and bootstrap tests were performed to assess indirect, direct, and total effects. As shown in Figure 2, both direct and total effects reached statistical significance (*p* < 0.05). Bootstrap analysis demonstrated a direct effect of HOMA‐IR at −0.648 (95% CI, −1.015–−0.282), with a total effect of −0.743 (95% CI, −1.106–−0.379) (Figure 2). The indirect effect mediated through the DTI‐ALPS index was estimated at −0.094 (95% CI, −0.236–−0.006), accounting for 12.66% of the total effect (Supplementary Table 1). It suggested that HOMA‐IR affects the DTI‐ALPS index, which in turn affects cognitive function.

### 3.4 The Mediating Role of Brain Glymphatic Function in the Relationship Between Insulin Sensitivity and Cognitive Domains

**FIGURE 3:**
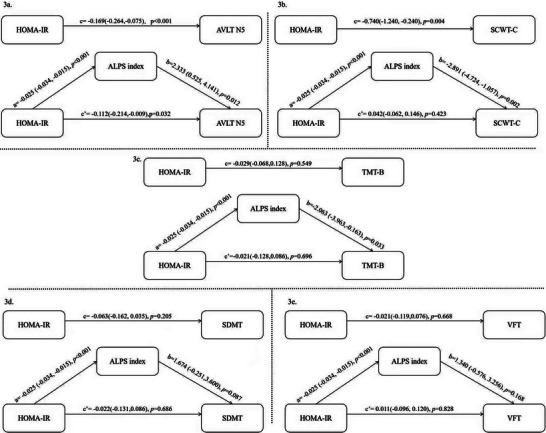
The mediating role of brain glymphatic function in the relationship between HOMA‐IR and cognitive domains. (a) The mediating role of brain glymphatic function in the relationship between HOMA‐IR and verbal memory (AVLT‐ N5), (b) the mediating role of brain glymphatic function in the relationship between HOMA‐IR and executive function (SCWT‐C), (c) the mediating role of brain glymphatic function in the relationship between HOMA‐IR and executive function (TMT‐B), (d) the mediating role of brain glymphatic function in the relationship between HOMA‐IR and attention function (SDMT), and (e) the mediating role of brain glymphatic function in the relationship between HOMA‐IR and language function (VFT). Abbreviations: AVLT, auditory verbal learning test; DTI‐ALPS index, diffusion tensor imaging along the perivascular space index; HOMA‐IR, homeostatis model assessment‐insulin resistance; MMSE, Mini‐Mental State Examination; SCWT‐C, stroop color‐word test‐C; SDMT, symbol digit modalities test; tCSVD score, total cerebral small vessel disease score; TMT‐B, trails making test‐B; VFT, verbal fluency test.

Figure 3 demonstrates the results of the mediation analysis of the DTI‐ALPS index between HOMA‐IR and various cognitive domains after controlling for covariates. In the total effects regression, HOMA‐IR was a significant predictor of verbal memory (B = −0.169, 95% CI, −0.264, −0.075, *p* < 0.001). This relationship remained significant when the DTI‐ALPS index was considered (B = −0.112, 5% CI, −0.214, −0.009, *p* = 0.032) (Figure 3a). The Sobel and bootstrap tests demonstrated indirect effects was −0.058, representing 34.32% of the total effect. As for attention, executive and language function, HOMA‐IR showed no significance with them (Figure 3b–e).

## 4 Discussion

By systematically examining the interplay among insulin sensitivity, glymphatic function (DTI‐ALPS index), and cognition in CSVD, we report three principal findings. (1) Relative to non‐IR subjects, IR patients exhibited a heavier CSVD burden, lower DTI‐ALPS index, and poorer global cognition as well as deficits in verbal memory, and executive function. (2) HOMA‐IR remained independently and inversely associated with the DTI‐ALPS index after adjustment for age, hypertension, CSVD burden, and other covariates. (3) Both the HOMA‐IR and DTI‐ALPS index predicted global cognitive performance; moreover, the DTI‐ALPS index significantly mediated the association between HOMA‐IR and global cognition (including the verbal‐memory domain) even after correction for multiple comparisons.

Accumulating evidence positions endothelial dysfunction as the core driver of CSVD pathology (Kremer et al. 2025). IR, the metabolic hallmark of systemic metabolic syndrome, amplifies this injury by inhibiting endothelial nitric‐oxide signal, amplifying oxidative stress, and sustaining low‐grade inflammation (Nakhaee et al. 2024; Petersen and Shulman 2018). These cascades jointly compromise BBB integrity, promote plasma extravasation, and accelerate small‐vessel arteriosclerosis, yielding the lacunar infarcts and WMHs that define CSVD neuroimaging (Kremer et al. 2025). Beyond structural damage, BBB leakage allows neurotoxic solutes to accumulate in the parenchyma, provoking mitochondrial failure, synaptic loss, and erosion of cognitive reserve (Montagne et al. 2017; Nakhaee et al. 2024). Our data extend these observations: participants with IR exhibited a three‐fold higher probability of a total CSVD score > 3 and a dose‐dependent relationship between HOMA‐IR and global cognitive impairment after adjustment for age, hypertension, and CSVD burden. These findings were in line with recent multicenter reports linking IR to both imaging severity and cognitive decline in CSVD cohorts (Cui et al. 2022; Yang et al. 2019). Collectively, the evidence frames insulin not merely as a metabolic hormone but as an endocrine guardian of cerebrovascular and synaptic health (Neth and Craft 2017).

The brain glymphatic system, a newly discovered perivascular fluid transport network, performs a crucial role in the brain's waste clearance mechanism. Pulsations of the cerebral arterial wall propel CSF through aquaporin‐4 (AQP‐4) channels on astrocytic endfeet into the PVS and subsequently into the brain parenchyma. This influx of CSF drives interstitial fluid and metabolic waste along perivascular routes toward the perivenous spaces surrounding the deep medullary veins, facilitating their clearance. The glymphatic system was influenced by multiple factors, including AQP‐4 expression, sleep status, and vascular pulsatility (Iliff et al. 2013; Szczygielski et al. 2021; Xie et al. 2013), but other risk factors remain to be fully elucidated. In our study, we found that the insulin sensitivity, HOMA‐IR, was an independent risk factor for the DTI‐ALPS index. This result aligns with a Japanese study in which metabolic syndrome (IR surrogate) was associated with a 0.42‐SD reduction in the DTI‐ALPS index after adjustment for age, sex, and white‐matter lesion load (Andica et al. 2023). In addition, clinical studies also demonstrated that impairment of the brain glymphatic clearance function was associated with increased severity of diabetes (Yang et al. 2020). Animal data corroborate the clinical observation: type‐2 diabetic rats display 30% slower hippocampal clearance than controls (Jiang et al. 2017), while chronic hyperinsulinemia shifts AQP‐4 from polarized end‐feet to a diffuse membrane distribution via mTOR–S6K‐mediated phosphorylation, reducing CSF influx (Petersen and Shulman 2018). Parallel work shows that IR uncouples endothelial nitric‐oxide synthase, attenuating vascular pulsatility and thereby weakening the “perivascular pump” that propels glymphatic flow (Nakhaee et al. 2024). Thus, IR appears to impair glymphatic efficiency through both molecular (AQP‐4 mis‐localization) and biomechanical (reduced pulsatility) mechanisms.

In turn, glymphatic failure may translate into cognitive vulnerability. We found the DTI‐ALPS index was associated with cognitive function, including overall cognitive function, verbal memory, and executive function in nondiabetic patients with CSVD, the results were consistent with an elderly study from China (Wang et al. 2023). The study, which included 633 participants, has presented that the DTI‐ALPS index was positively associated with cognitive function cross‐sectionally (B = 0.108, *p* = 0.003). Longitudinal work from the UK CSVD program further showed that the baseline ALPS index halved the 7‐year dementia risk among lacunar stroke patients with confluent white‐matter lesions (Hong et al. 2024). Mechanistically, CSVD‐induced arteriosclerosis—marked by small‐arterial wall thickening and luminal narrowing—dampens arterial pulsatility, thereby weakening the motive force that powers CSF–interstitial fluid exchange and slowing glymphatic flux (Iliff et al. 2013). Concomitant chronic hypoperfusion depolarizes astrocytic AQP‐4, impeding CSF entry into the parenchyma and further compromising metabolic‐waste clearance (Dong et al. 2024; Szczygielski et al. 2021). Pre‐clinical studies show that accelerating glymphatic clearance reverses memory deficits (He et al. 2020; Liu et al. 2020), establishing the system as a tractable therapeutic target. In our cohort, mediation analysis revealed that the DTI‐ALPS index explains 12.7% of the HOMA‐IR–cognition link after adjustment for age, education, and global CSVD burden. This carries dual translational weight: (1) ALPS may outperform conventional CSVD scores as an early marker because it gauges waste‐clearing capacity rather than structural damage alone; (2) enhancing glymphatic function could interrupt the causal cascade from insulin resistance to cognitive decline. Mediation analysis in our sample indicated that the ALPS index conveys 12.7% of the total effect of HOMA‐IR on global cognitive impairment—a proportion that remained stable after adjustment for age, education, and MRI burden scores. This implies that glymphatic dysfunction is not merely an epiphenomenon of vascular injury but acts as a quantifiable and potentially modifiable pathway linking IR to cognitive decline. From a translational perspective, the ALPS index may serve as an early imaging biomarker that captures metabolic‐waste accumulation before irreversible structural damage accrues, while interventions that restore insulin sensitivity could concurrently rescue glymphatic flux and preserve cognitive reserve in CSVD.

The present study has some limitations. First, our cross‐sectional study had a small sample size with an unequal number of participants between groups that might have limited the statistical power, while recruiting healthy older adults was indeed a challenge for this current research. Future studies should be conducted on longitudinal data of larger population samples with a balanced proportion. Second, the use of MRI‐based tracers was considered the current gold standard for measuring glymphatic function in humans (Taoka and Naganawa 2020), which was not applied in our study. However, as mentioned previously, the DTI‐ALPS index was highly correlated with glymphatic clearance function, as calculated on glymphatic MRI after intrathecal administration of gadolinium (Zhang et al. 2021) Given its non‐invasiveness, the DTI‐ALPS index has been widely adopted in clinical practice.

## 5 Conclusion

Our study establishes HOMA‐IR and the DTI‐ALPS index as independent determinants of cognitive performance in non‐diabetic CSVD and, for the first time, reveals that the DTI‐ALPS index significantly mediates part of the HOMA‐IR–cognition pathway. These findings illuminate how metabolic perturbations disrupt the “vascular–neural–cognitive” axis, positioning improved insulin sensitivity and augmented glymphatic flux as a combined, mechanistically grounded strategy for cognitive protection in CSVD. Large‐scale, multi‐center intervention trials are now warranted to confirm causality and guide clinical implementation.

## Author Contributions

Conceptualization: Xiao‐Li Yang and Yu‐Fan Luo. Data curation: Meng‐Xiang Wang and Wen‐Mei Lu. Formal analysis: Tian‐Yao Wang. Methodology: Xiao‐Li Yang and Yu‐Fan Luo. Project administration: Dan‐Hong Wu and Hu‐Lie Zeng. Visualization: Yu‐Fan Luo. Writing – original draft: Xiao‐Li Yang. Writing – review and editing: Dan‐Hong Wu and Hu‐Lie Zeng.

## Funding

This study was supported by grants from the Minhang District Natural Science Foundation(2024MHZ036), Shanghai Minhang District Health and Family Planning Commission for Constructing Big Disciplines (2024MWDXK04), and Health Profession Clinical Research Funds of Shanghai Municipal Health Commission (202540025).

## Ethics Statement

This study was approved by the regional ethical committees of Shanghai Fifth People's Hospital (approval number: 2021–211). Written informed consent was obtained from all participants or authorized representatives. All research procedures adhered to the tenets of the Declaration of Helsinki.

## Supporting information




**Supplementary Material**: brb371143‐sup‐0001‐SuppMat.docx

## Data Availability

The datasets used and /or analyzed during the current study are available from the corresponding author upon reasonable request.
