# Supplementary material for: Insulin Resistance and Cognitive Function in Nondiabetic Patients With Cerebral Small Vessel Disease: Role of Brain Glymphatic Function
Source: Brain Behav. 2025 Dec 22;15(12):e71143. doi: 10.1002/brb3.71143 (PMC12723189; doi:10.1002/brb3.71143)
Supplement: Supplementary file 1 — Supplementary Material: brb371143‐sup‐0001‐SuppMat.docx [file BRB3-15-e71143-s001.docx]

**Supplementary Methods**

**Magnetic resonance imaging (MRI) protocol**

Cerebral small vessel disease (CSVD) patients received MRI examinations on a GE 3.0-T scanner (Discovery MR750, GE Healthcare, Milwaukee, WI, USA) with a standard 8-channel HRBRAIN coil. The full sequence of cranial MRI included axial 3DT1-weighted sequences, T2-weighted sequences, T2 fluid-attenuated inversion recovery (FLAIR), susceptibility-weighted imaging (SWI) and diffusion tensor imaging (DTI) sequences. The MRI parameters are as follows: (1) 3DT1 -weighted sequence: Time of acquisition (TA)=5:45, repetition time (TR)=2400ms, echo time (TE)=2.98ms, (FOV)=256mm, slice thickness=1mm, Voxel size: 1.0×1.0×1.0mm, Slices per slab=192, Inversion time (TI)= 1100ms, Flip angle=7deg. (2) T2-FLAIR sequence: TR=9000ms, TE=85ms, Voxel size: 0.5×0.5×6.0 mm, Slices=20, FOV=240 mm, slice thickness=6.0mm with no gap between slices, Flip angle=150deg. (3) SWI sequence: TR=28ms; TE=20ms; FOV=240mm; slice thickness=1.5mm, Voxel size: 0.3×0.3×1.5mm, Flip angle=15 deg. (4) DTI sequence: TA: 6:30, Voxel size: 2.0×2.0×2.0 mm, FOV=224 mm, Slice thickness=2mm, Slices=75, TR=10800ms, TE= 90.0ms, Maximum b-value=1000s/mm2, 30 non-collinear directions.

**Supplementary Figure 1.** **Diffusion tensor image (DTI) analysis along the perivascular space (DTI-ALPS) workflow.**

**
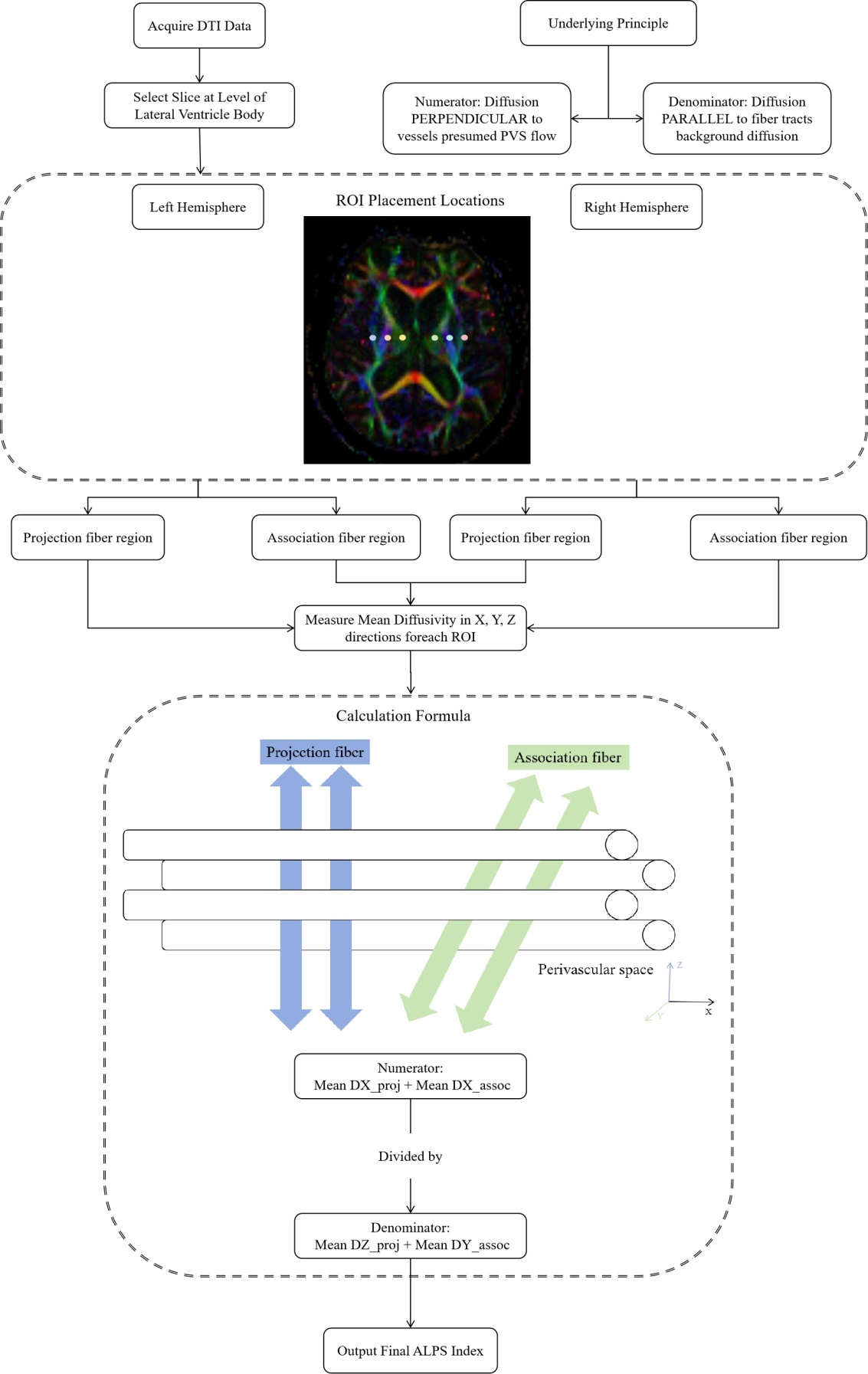
**

**Supplementary Figure 2. Flow diagram of the inclusion process**


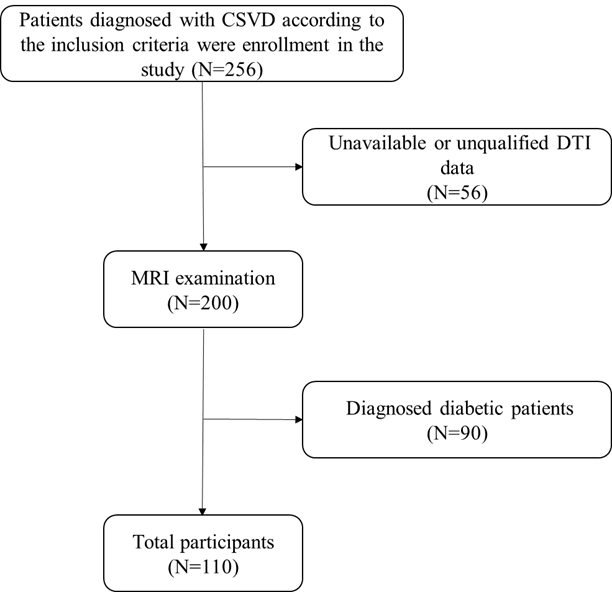


**Supplementary Table 1. Sobel and Bootstrap tests for mediation models**

| MMSE |  | Effect | p | LLCI | ULCI |
| --- | --- | --- | --- | --- | --- |
|  | Total effect | -0.743 | <0.001 | -1.106 | -0.380 |
|  | Direct effect | -0.649 | <0.001 | -1.015 | -0.282 |
|  | Indirect effect | -0.094 | 0.029 | -0.236 | -0.006 |
